# Artificial Intelligence-Assisted Online Social Therapy for Youth Mental Health

**DOI:** 10.3389/fpsyg.2017.00796

**Published:** 2017-06-02

**Authors:** Simon D'Alfonso, Olga Santesteban-Echarri, Simon Rice, Greg Wadley, Reeva Lederman, Christopher Miles, John Gleeson, Mario Alvarez-Jimenez

**Affiliations:** ^1^Orygen, The National Centre of Excellence in Youth Mental HealthMelbourne, VIC, Australia; ^2^School of Computing and Information Systems, The University of MelbourneMelbourne, VIC, Australia; ^3^Centre for Youth Mental Health, The University of MelbourneMelbourne, VIC, Australia; ^4^Faculty of Education Sciences and Psychology, Universidad Rovira i VirgiliTarragona, Spain; ^5^School of Psychology, Australian Catholic UniversityMelbourne, VIC, Australia

**Keywords:** youth mental health, psychosis, depression, computational health, chatbots, sentiment analysis

## Abstract

**Introduction:** Benefits from mental health early interventions may not be sustained over time, and longer-term intervention programs may be required to maintain early clinical gains. However, due to the high intensity of face-to-face early intervention treatments, this may not be feasible. Adjunctive internet-based interventions specifically designed for youth may provide a cost-effective and engaging alternative to prevent loss of intervention benefits. However, until now online interventions have relied on human moderators to deliver therapeutic content. More sophisticated models responsive to user data are critical to inform tailored online therapy. Thus, integration of user experience with a sophisticated and cutting-edge technology to deliver content is necessary to redefine online interventions in youth mental health. This paper discusses the development of the moderated online social therapy (MOST) web application, which provides an interactive social media-based platform for recovery in mental health. We provide an overview of the system's main features and discus our current work regarding the incorporation of advanced computational and artificial intelligence methods to enhance user engagement and improve the discovery and delivery of therapy content.

**Methods:** Our case study is the ongoing Horyzons site (5-year randomized controlled trial for youth recovering from early psychosis), which is powered by MOST. We outline the motivation underlying the project and the web application's foundational features and interface. We discuss system innovations, including the incorporation of pertinent usage patterns as well as identifying certain limitations of the system. This leads to our current motivations and focus on using computational and artificial intelligence methods to enhance user engagement, and to further improve the system with novel mechanisms for the delivery of therapy content to users. In particular, we cover our usage of natural language analysis and chatbot technologies as strategies to tailor interventions and scale up the system.

**Conclusions:** To date, the innovative MOST system has demonstrated viability in a series of clinical research trials. Given the data-driven opportunities afforded by the software system, observed usage patterns, and the aim to deploy it on a greater scale, an important next step in its evolution is the incorporation of advanced and automated content delivery mechanisms.

## Introduction

The majority of early intervention programs (specialist interventions and support to young people experiencing early symptoms of mental illness) offer services that do not last more than 2 years; for instance, early intervention services for psychosis typically offer support for 24 months. headspace, an Australian national foundation that provides early intervention mental health services to young people offers up to only 10 sessions of psychological therapy per year (Rickwood et al., 2014, 2015). As a result, some of the benefits gained through specialized treatment may not persist after its termination. Discharge and referral to a general mental health service may create a feeling of detachment among young people, decreasing engagement with mental health institutions and consequently increasing chances of relapse. In fact, reviews indicate that up to 80% of young people relapse from their initial condition after symptomatic remission from psychosis or depression (Alvarez-Jimenez et al., 2012).

Recent years have seen the development of online interventions to address these issues. Novel information and communication technologies provide an extraordinary opportunity for improving, and even transforming interventions with psychiatric disorders (Álvarez-Jiménez et al., 2016). Due to the general enthusiasm of young people for new technologies, with more than 97% of youth connecting to the Internet daily (Pew Research Center, 2014), internet-based interventions may be especially effective for, and attractive to, patients with different mental health disorders (Burns and Morey, 2008). Pioneering interventions using these technologies may play a pivotal role in addressing substantial challenges, comprising access to and engagement with services, and delivery of extended support to maintain the clinical gains of specialized services (Álvarez-Jiménez et al., 2012). Internet-based interventions may lead to the development of supportive relationships (O'Keeffe and Clarke-Pearson, 2011), decreased isolation (Dennis, 2003), increased self-disclosure (Weisband and Kiesler, 1996), and may possibly reduce stigma (Houston et al., 2002).

Social networking interventions in particular are uniquely placed to support young people experiencing mental ill health (Rice et al., 2014). Due to the stigma associated with mental illness, young people experience extreme social isolation and face difficulties in maintaining relationships (Morgan et al., 2011). The use of online social networking sites has been associated with positive socialization, promotion of supportive relationships, increased self-esteem, facilitation of communication, and feelings of group membership among young people, underscoring the relevancy among adolescents at risk of social isolation (Collin et al., 2011; O'Keeffe and Clarke-Pearson, 2011). These benefits may create a sense of belonging for young people, increasing use and engagement with social networks on the Internet.

The moderated online social therapy (MOST) project (Alvarez-Jimenez and Gleeson, 2012; Gleeson et al., 2012; Lederman et al., 2014) is designing, building and testing online social therapy systems for youth mental health. The MOST model uniquely integrates online peer support and evidence-based interventions with a clinician and consumer-centered service delivery process. It has also been developed following participatory design principles and it uses persuasive design elements to promote engagement with the intervention and behavioral change (Hagen et al., 2012).

To date, the MOST model has been effectively implemented in six studies, including four pilot studies; (i) Horyzons, for young people recovering from psychosis (Alvarez-Jimenez et al., 2013), (ii) Momentum, for young people at ultra-high risk of developing psychosis, (iii) Rebound, for young people recovering from depression (Rice et al., 2016), (iv) Meridian, for carers looking after young people experiencing mental health issues. In addition, there are currently two active longer-term randomized controlled trials evaluating MOST, (v) as a 5-year relapse prevention intervention for first episode psychosis (the Horyzons RCT), and (vi) a 2-year trial to support carers of young people with psychosis (the Altitudes RCT). Generation is also an upcoming trial in collaboration with eheadspace^1^ (Rickwood et al., 2014, 2015) using the MOST software to power a general site for help seeking young people.

The MOST project was initiated with the aim of investigating two questions:

1. Efficacy of online therapy: do the clinical benefits of specialized face-to-face youth mental health programmes extend into long-term improvements through online psychosocial intervention?
2. Technology design: how can we best design and implement engaging technology in for young people with mental-ill health?

Recently a third question has arisen.

3. How can advanced computational and artificial intelligence (AI) methods be employed to supplement the support provided by moderators/clinicians and automate user-tailored therapy with a view to scaling up usage of the MOST model and platform?

Usage analyses of the MOST sites indicate that the social networking component is the most frequently used feature (Rice et al., 2016). Despite these favorable system usage statistics, we strive to improve the delivery of therapeutic aspects of the system to our users. Thus, this paper focuses on some recent research and incipient developments pertaining to the third question. We use the Horyzons site as our example, since it is the longest running implementation and has the largest set of usage data.

## Accessing site content

A large focus of development efforts has been on ways in which online therapy content within MOST can best be delivered to users so that it is relevant, draws their interest and maximizes their engagement. MOST follows a positive psychotherapy model (i.e., strengths-based models; Seligman et al., 2006) and a theory-driven model of online human support by moderators (i.e., supportive accountability; Mohr et al., 2011). The creation of therapy content in the MOST sites was driven by feedback from users and expert youth mental health clinicians through iterative prototyping and participatory design. The software system was designed via a tailored (bespoke) design process, which offered more flexibility to integrate social networking, therapy and the moderation component (Wadley et al., 2013). The result is a therapeutic environment where young people can learn and practice therapeutic techniques, gain perspective and validation, and learn how to solve problems in a transitional social network on their path toward recovery.

**Figure 1:**
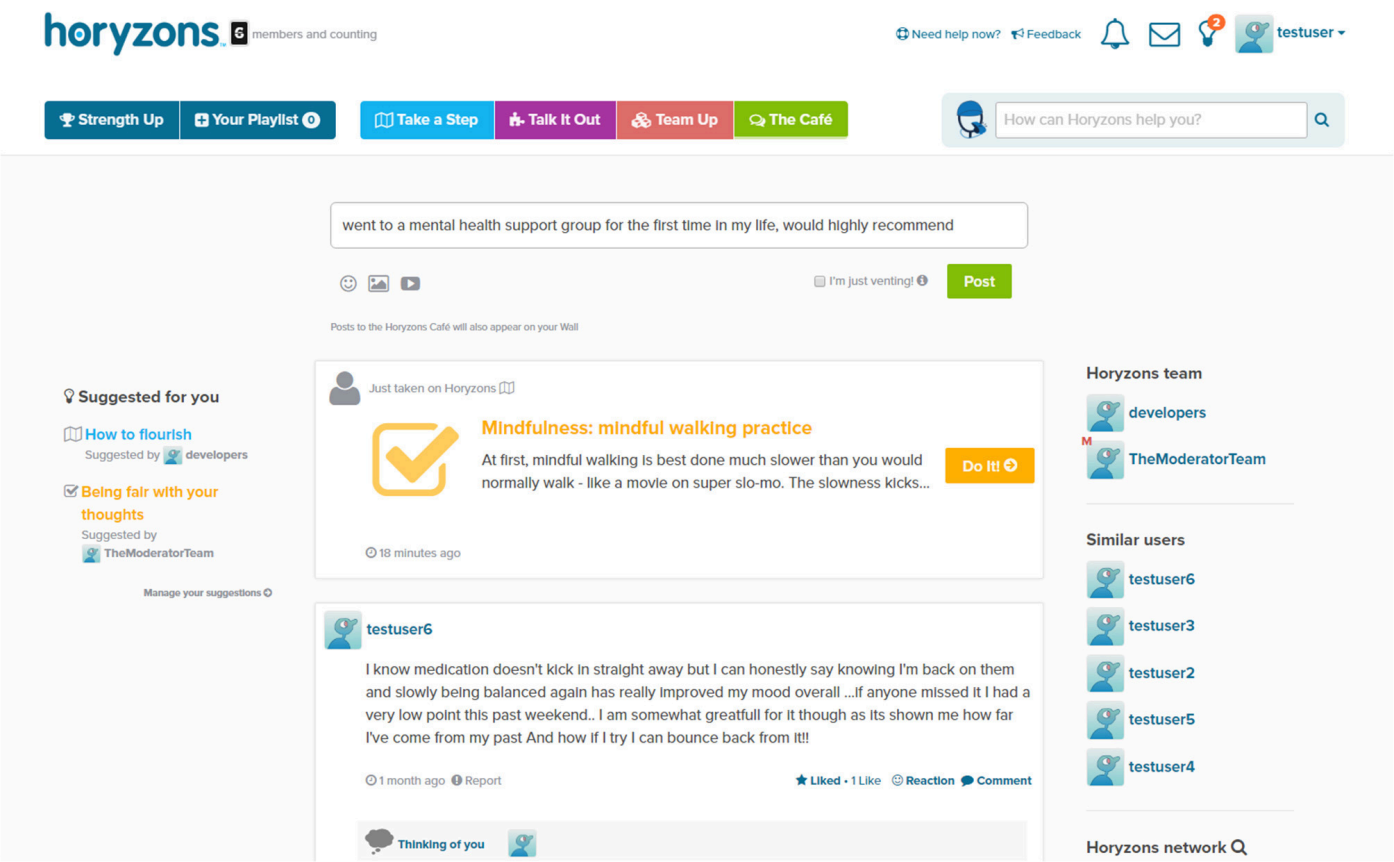
**The Horyzons Cafe newsfeed**.

**Figure 2:**
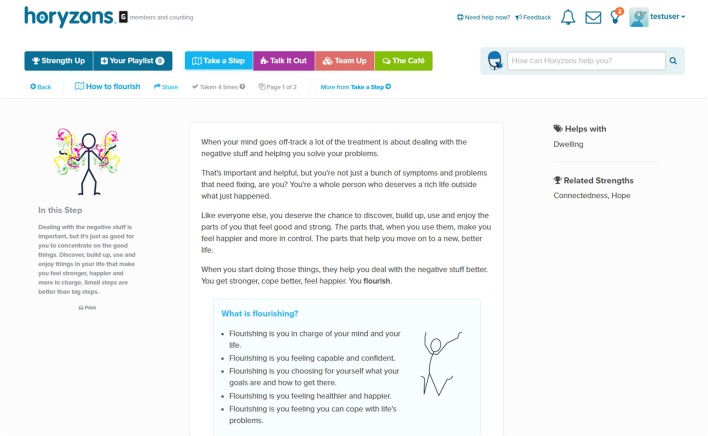
**The beginning of a step on Horyzons**.

**Figure 3:**
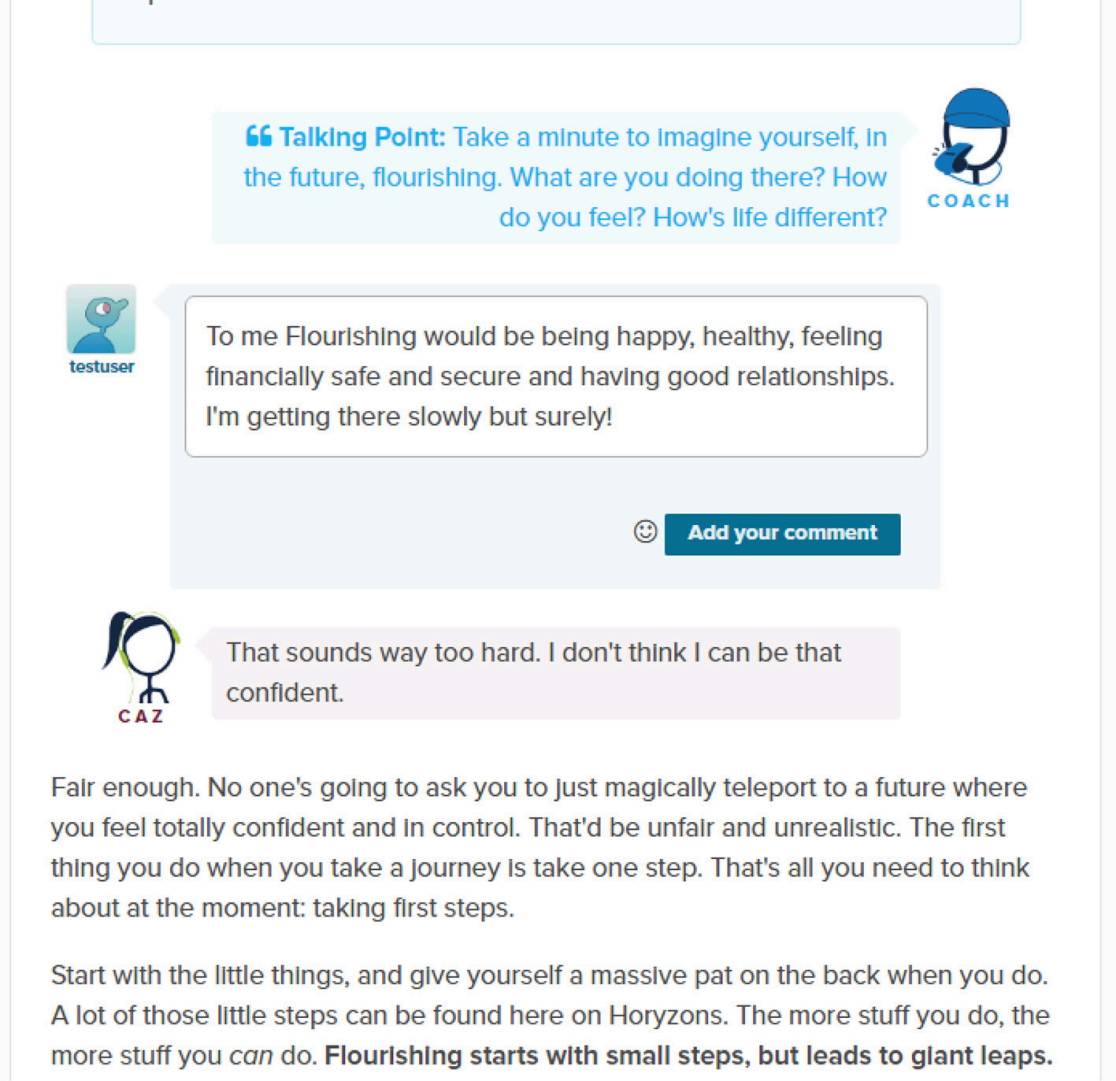
**The Talking Point in the How to Flourish step**.

**Figure 4:**
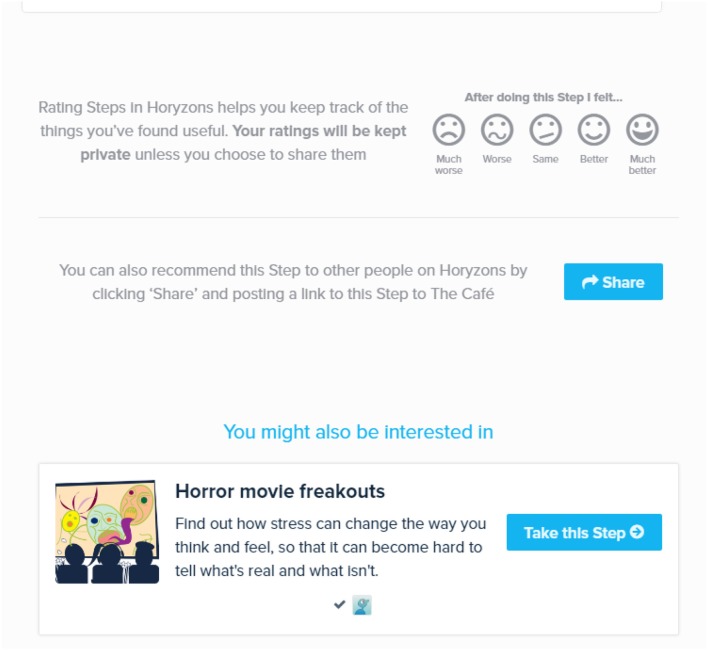
**Finishing the step How to Flourish**.

The modules were designed in a collaborative effort between professional writers for young people, clinicians, psychology researchers, and users (Lederman et al., 2014). Following is an outline of the MOST system's main parts:

1. *Social Networking and The Cafe*—A Facebook-style newsfeed where users can contribute posts and comments; share experiences, give and obtain support, and gain perspectives and validation. Additional features include a *Job Zone* for vocational opportunities and information and *Team Up*, where users can set a challenge for themselves that others can then also participate in or follow as part of a “cheer squad.” See Figure 1 for an illustration.
2. *Take a Step*—Steps are interactive therapy modules designed to exercise and develop a range of psychological skills. Therapy is delivered via engaging content that has been developed collaboratively by clinical psychologists, professional creative writers, leading comic developers, and young people. Social interaction is embedded within steps through *Talking Points*, which are questions that promote users to discuss and share their own experiences. See Figures 2, 3, 4 for illustrations.
3. *Talk It Out*—A space where users can nominate problems or difficulties they would like some help with to discuss in moderated groups following an evidence-based social problem solving framework (McFarlane et al., 1995; McFarlane, 2004). Once a user has nominated a problem and framed it together with a moderator, solutions are proposed and discussed by the users before a moderator wraps up the thread with a synopsis. To date there are 75 completed Talk It Outs. Apart from the role each of these played in providing an active forum to address a user-nominated problem, the overall result is a valuable user generated “knowledge base” repository that can be searched and referred back to.
4. *Do It! (Actions)*—Users can access and “do” specific behavioral experiments or tasks (referred to as “actions”) to apply mindfulness, self-compassion, and personal strengths in real world situations relevant to the young person (e.g., social context, school, work, alone, etc.). The use of context specific, action-based suggestions through online interventions has been recommended to change behavior, develop skills and increase practice, and generalization of these skills to real life situations (Van Gemert-Pijnen et al., 2014). Users can also bookmark actions so that they can easily return to and periodically reengage with those that they find helpful.

Conceptually, we can distinguish between The Cafe, a social space generated by user contributions and the Take a Step and Do It! sections, which offer authored therapeutic content for users to engage with. The Talk It Out section lies somewhere in between; Talk It Outs are generated by user contributions and the result is a valuable repository of material that users can access. Importantly, the MOST system also integrates these sections and functions; therapy content appears throughout the newsfeed and micro newsfeed-like discussion threads can occur in therapy pages. In general, integration is key and the system has been designed to create a constant back and forth flow for the user between therapy and social elements.

For example, suppose that a user starts on the newsfeed café page (Figure 1). They can comment on and react to posts from other users or they can add their own posts^2^. As will soon be covered in more detail, they also have access points to therapy content; moderator suggestions appear to the left, posts advertising content just taken by others users appear in the newsfeed and therapy suggestions relative to a user's own posts are made upon their submission. Suppose they click on the “How to flourish” step (Figure 2). While going through the step, they can interact with the step and contribute to a “mini-newsfeed” by commenting on a Talking Point (Figure 3). Upon completing the step, they can feed back into The Café by sharing a link to the step or rating the step and sharing this rating with an accompanying message. Also, a recommender system suggests other relevant steps the user might like to try (Figure 4).

## Discovering content

The most basic and direct ways in which users can access steps and actions are via a simple omnipresent search bar and the aforementioned primary navigation menu links. The ubiquity of search bars in this “Age of Search” make them a natural interface element familiar to Internet users. At present the search function accepts a simple search term and performs a basic text match against content in the system. Interestingly, it is not used a great deal and is used more so to search for users and site features rather than therapy content. We are currently looking into revamping this interface real-estate. Ultimately an “oracular” search box that performs a more sophisticated, customized form of information retrieval could process expressive input from the user and instantly provide a gateway to relevant therapy content.

**Figure 5:**
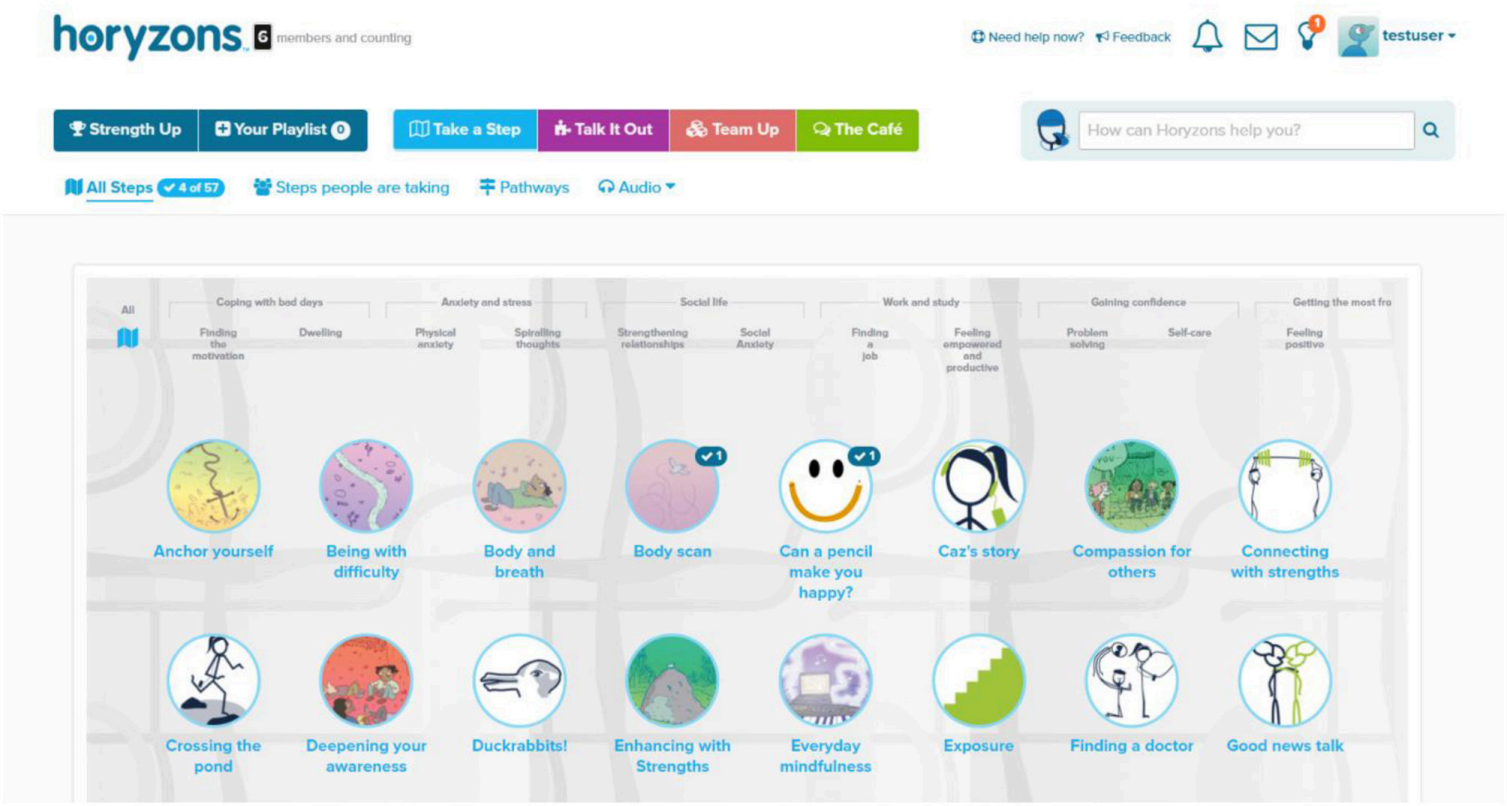
**Part of the All Steps page, where users can alphabetically browse steps**.

Under the steps section users can browse an alphabetically-ordered grid of all the steps presented in a visually appealing manner with respective icons, as illustrated Figure 5.

Given the potential for users to be encouraged to participate based on the activity of their peers, we also include a page “Steps People are Taking.” The four access links featured on this page are:

1. Steps taken recently—Among all the steps visited from these four options, 48% of the visits resulted from this link.
2. Steps that have got people talking—Users are presented with the opportunity to comment on Talking Points at various points during steps. This option selects those steps with the most Talking Point comment activity. Accounts for 14% of all step visits.
3. Steps taken most often—Accounts for 11% of all step visits.
4. Hidden Treasures—A modest means of countering informational cascades (Easley and Kleinberg, 2010) and promoting less popular steps by displaying those steps that have been done the least. The euphemism seems to have been effective, as this link accounts for 27% of all step visits from these four options.

Actions have a similar interface and users can also engage with actions in the “Powered by your Strengths” page. Upon joining the system, users can complete an initial exercise where they choose 5 out of 24 strengths that they believe best apply to them. Some example strengths are Courage, Discretion, Creativity, and Curiosity. Strengths are connected to relevant actions through which they can be exercised and this option simply presents users with actions that are connected to the strengths they have chosen.

Whilst the presence of these structures provides a straightforward means to access content, their supplementation with more advanced, automated and user-tailored options is desirable. The following considerations concerning the limitations of these standard access options serve as a starting point for the justification of such supplementation. Search bars are a simple, effective means to find content. Sometimes though a user might not have the right search term in mind for what they are seeking or they might not have the intention to seek. Also, it could be that their behavior on the site suggests that they could benefit from something they are not even aware of. Direct menu links are good for users who are keen to browse the therapy content or for those who have the motivation to search for something they have in mind. This will often not be the case though and we cannot always expect this from users. Thus, rather than expecting users to always seek content or know what they want to search for, we endeavor to develop more sophisticated forms of content delivery. As a starting point for this endeavor, we believe that users may benefit from more advanced taxonomic guidance in finding relevant content. This consideration led to the incorporation of a tag-based system.

## Therapy tags

The purpose of tags is to create meaningful categories based on therapeutic content, which addresses target groups of symptoms (i.e., anxiety) or skills (i.e., social abilities). This classification allows both users and clinicians to find or suggest individually-tailored content for each person, taking into account the particular emotional state and their needs at that precise moment. There are three parent tags in the system and each of these has three sub-tags:

- Coping
  ◦ Solving Problems
  ◦ Kicking the Habit
  ◦ Beating Sadness and Worries
- Connecting
  ◦ Overcoming Conflict
  ◦ Boosting Relationships
  ◦ Stories Like Yours
- Enhancing
  ◦ Making Happiness
  ◦ Wellbeing with Mindfulness
  ◦ Work and Study

Steps and actions are tagged with one or more of these sub-tags and users can accordingly view or search for this content via these tags.

## Human-supported engagement

Apart from providing navigational/taxonomic structures to access therapy content, the MOST system also involves the direct delivery of specific content suggestions to individual users. From its inception, a feature of the MOST system has been that moderators can select content to suggest to users. Moderation by staff has been essential to increase adherence, because positive user motivation is enhanced by accountable and trusted experts who moderate site usage (Mohr et al., 2011). Moderators not only provide safety by preventing misuse of the system, but encourage usage and enhance user experience by acting as a role-model. The moderation team includes seven clinical psychologists and a clinical social worker. Moderation of specific topics is provided by a vocational worker and an expert in youth participation. Moderator therapy suggestions are based on what the user's moderator deems to be appropriate based on knowledge of factors such as the user's history and profile, as well as their current engagement with and activity on the site. For example, one of our moderators was informed via discussion with a user that the user had recently gained employment. With knowledge of both this and the fact that the user had not yet chosen any strengths, the moderator suggested the step “*Strengths for work*,” which helps users to find ways to use their strengths to survive and thrive at work. If a suggested content item is completed by the user, then the system automatically records this. There are also the options of a client or a moderator dismissing the suggestion. Naturally, such information helps to inform subsequent moderator suggestion choices.

The main advantage of moderator suggestions is that they are customized based on expert moderator assessments of an individual user and what therapy content is considered appropriate for them. The personalized suggestion perhaps makes the user more receptive to engagement with the content. To date, there have been a total of 701 moderator-to-user suggestions. Out of this number, 211 have been completed (21 have been client dismissed, 304 moderator dismissed, and 165 pending). This is actually a fairly successful strike rate relative to other therapy content access points in the system. Our content completion rates compare well with short-term Internet-based programs (Christensen et al., 2004), and also compared to online interventions comprising some kind of moderation by interviewers or counselors (Christensen et al., 2004; Clarke et al., 2005). Furthermore, of the various therapy content access points, moderator suggestions have the highest usage number; around 39% of all tracked visits to steps/actions are due to moderator suggestions.

## Automated suggestions

Whilst manual moderator suggestions are a key part of our current sites, we are investigating automated methods for content delivery and how they can be used to facilitate user engagement; complementing our current models and addressing limitations. The first obvious advantage of automated suggestions over moderator suggestions is that they are not limited to times when moderators are available on the system and furthermore they can be delivered to the user in real-time. Automated suggestions also facilitate scaling the site to a larger user group. At present, the MOST platform is used with relatively small groups of users in a research setting (numbers ranging between 30 and 100 as opposed to hundreds, thousands, or more). Each moderator is assigned a manageable list of users, which they monitor and attend to. Although, such automated therapy suggestion methods currently serve as an alternative rather than a replacement for moderator suggestions, given the aim of scaling up and deploying the MOST platform in a more general, less moderated, or even unmoderated publicly available setting, automation becomes a prime goal.

Originally, the only form of automated content suggestion occurred when a user did a step. In a manner similar to Amazon's related recommendations, upon completing a step, a list of related steps is dynamically presented to the user, preceded by the message “*You might also be interested in*.” Around 5% of all tracked step visits are from this path. Related steps are determined by step-to-step connections pre-set by the system author. The next subsequent additions involved inserting therapy suggestions into the newsfeed. Firstly, when a step/action is done, this fact is anonymously posted in the newsfeed. Secondly, we make select action suggestions into the newsfeed, in a manner similar to Twitter/Facebook feed ad placements. These suggestions are based on the user's chosen strengths and any steps they have taken. Around 9% of all tracked action/step visits are from this path.

These two features offer an easy to implement, cost-effective way to promote therapy content compared to the suggestions made by a moderator. Human presence is not needed, the user receives the suggestion immediately, and if they perform another activity, a new related suggestion will appear in their screen. However, their delivery is not necessarily user-tailored or immediately relevant. A recently incorporated feature addresses such issues and connects newsfeed posting activity with therapy content suggestions. The basic idea is that linguistic analysis of user posts can extract certain information on which to base content suggestion. As soon as a user submits a post, an algorithm sets off to analyse the post and determine one step, and one action, that are relevant to the post. The way these suggestions are presented has raised some usability issues and questions. Due to the calculations and remote application programing interface (API) calls (3Scale NSL, 2011) required, it can take up to 10 s in order to calculate these suggestions. This lag initially posed a significant problem from a user interaction perspective, as the calculation was made sequentially before the post was added to the newsfeed, leaving the users wondering what was happening. We therefore decided to calculate the suggestions parallel to the posting of the post. Upon addition of a post to the newsfeed, users receive the message “*Horyzons has suggestions based on your post*” above the post. If the user presses the “*Show Me*” button next to this message, the system attempts to retrieve the suggestions and display them above the post. If the suggestions have not been calculated yet, then the system displays a “*Delivering your suggestions*” message along with a dynamic progress image. It is hoped that this provides users with a sense that the calculation is very much occurring and it is occurring specifically in response to their posting activity. In order to foster a sense of privacy and individuality, we also accompany this with the message “These suggestions are only visible to you.”

With this suggestion delivery interface now determined, our focus is on developing and experimenting with the underlying algorithm that calculates the suggestion. At present, we use a combination of the post's sentiment, emotion scores, and keywords to determine its most relevant steps and actions. For the reader's interest, a brief description of our current algorithm is as follows:

1. Upon submission of a post to the newsfeed, a call is made to IBM's Alchemy [10] text analysis system. First, it provides a score for the post's sentiment, with score = 0 being neutral, 1 > score > 0 being positive, and score 0 > score > −1 being negative. Second, it provides a score between 0 and 1 for the emotions of Anger, Disgust, Fear, Joy, and Sadness. Third, it extracts the post's keywords^3^.
2. Our steps and actions have been partitioned into those which are suitable for positive posts, those which are suitable for negative posts and those which are suitable for either. We use this partitioning such that if a post is positive then a set of suggestion candidate steps/actions is reduced to those marked positive. Similarly for negative. If the post is neutral, then no such reduction is made.
3. Our steps and actions have also been assigned measures for each of the five emotions mentioned in point 1; the higher the measure, the more relevant the item is for posts exhibiting that emotion. We also have extracted and stored the keywords for all of our steps and actions. Given this, each of the candidate steps/actions is assigned a score based on their semantic similarity with the post's keywords and congruence with the post's emotion measures.
4. The step with the highest score is selected as is the action with the highest score. If more than one candidate emerges, at this stage one is randomly chosen.

The keyword semantic similarity component is the most decisive factor in these calculations. Given that much of our therapy content is specifically suited to only positive or negative states, the sentiment analysis component determines an important initial partitioning of therapy content where applicable. The emotion congruence component provides a balancing and adjudicating factor. First, it can distinguish therapy items that have the same semantic similarity score. Second, it can “downgrade” therapy items with a relatively high semantic similarity score that are otherwise emotionally inappropriate relative to the post.

**Figure 6:**
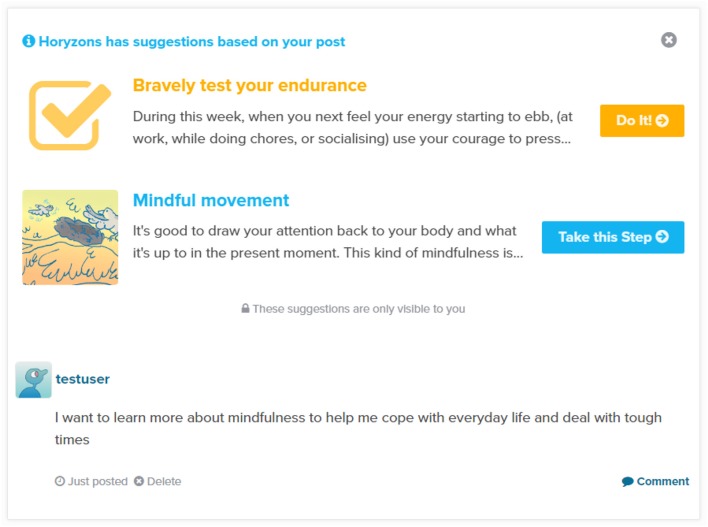
**An example of a step and action suggested in response to a user post**.

Figure 6 illustrates a simple example post and accompanying suggestions. As can be gathered from their titles, the suggested step and action are quite relevant relative to the post content.

**Figure 7:**
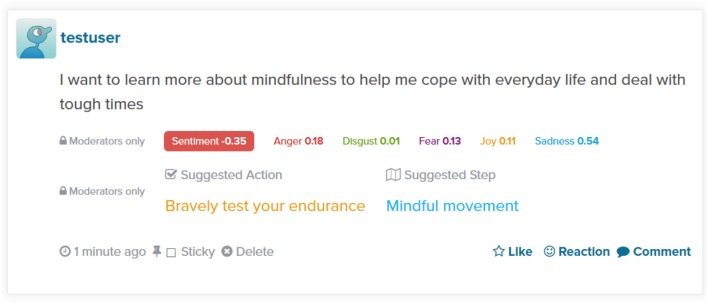
**Moderator view of a post**.

Once the page is reloaded these suggestions disappear. As Figure 7 illustrates, they do though remain visible to moderators, along with sentiment and emotion information.

This initial algorithm has just been implemented and we will soon be analyzing our records of suggestions that have been made in order to gain insight into how it can be improved. Ultimately, there are many linguistic/psychometric properties that could be of use in pairing posts with relevant therapy content (Tausczik and Pennebaker, 2010).

There are also plans to include a feedback interface next to the suggestions so that users can rate how relevant the suggestions were for them. This data will help to influence our algorithm and content scoring in general and will also provide us with information on how we can further tailor content to specific users. Apart from refining the custom selection of content suggestions, there is also the possibility of tailoring the message given to a user upon suggestion of a piece of therapy content. By analyzing a post and other historical information about the user, we could offer messages of the form “This step was recommended to you because ….” Beyond using and responding to single posts, there is also the possibility of using larger segments of user input accumulated over time. Along with other pertinent user usage data, these segments could help to determine periodic automated suggestions akin to moderator suggestions that are delivered independently of and not in response to a specific user action on the site. Finally, it should be noted that statistics provided over the last few sections are used to make comparisons between the ways in which site content can be accessed; how people are navigating the site and their preferred methods for accessing content. We are yet to conduct any A/B or split testing to evaluate the impact of site features, although this is something that is on our agenda. Before closing, we will next briefly look at the possibility of incorporating chatbots into online social therapy.

## Chatbots

A chatbot is a computer program that mimics conversation with users via a chat interface, either text or voice based (Abdul-Kader and Woods, 2015). The underlying system can be based on a variety of foundations, ranging from a set of simple rule-based responses and keyword matching to powerful natural language processing (NLP) (Chowdhury, 2003) and machine learning (ML) (Smola and Vishwanathan, 2008) techniques; NLP concerns the use of computers to understand and manipulate natural language and ML concerns self-learning computer programs with the ability to grow and adapt in response to new data, without being explicitly programmed to do so (Saeed, 2016). Irrespective of the actual intelligence of the responding bot, there is something distinct about the experience of a user entering input and a bot responding. A bot may create the sensation of a natural and real environment for the user since it can understand natural speech patterns. While an app or a web search may give the user a direct answer in response to a search query, a bot simulates a real-life conversation as if the user was talking with another person; the uniqueness of the feature resides in the user's perception of an interaction (Margalit, 2016). Also, beyond the challenge of imbuing chatbots with intelligence in terms of their ability to simulate the structures of natural language communication, another important dimension, particularly in a psychology/therapy setting, is emotional intelligence; developing chatbots that can detect and respond appropriately to the emotional state of the human. Some recent work on emotionally intelligent AI comes from the province of affective computing^4^ (Brewster, 2016; Skowron et al., 2017).

Chatbots are currently a hot topic in the tech world, with major technology companies such as Facebook, Microsoft, and Google making significant investment forays into this emerging technology. The commercial applications of chatbots range from the provision of online customer service to conversation-based product searches and event organization. Whilst our motivations differ, this current commercial interest in chatbots makes for a particularly opportune time to consider their incorporation into online mental health platforms such as MOST. In fact, the history of chatbots is intimately tied with psychology. Apart from the interesting philosophical and psychological questions they raise, the first well-established chatbot, ELIZA was actually programmed (in 1966) to simulate a Rogerian psychotherapist (Weizenbaum, 1966). A modern ELIZA, the more sophisticated Artificial Linguistic Internet Computer Entity (A.L.I.C.E.), first surfaced in 1995 (Shah, 2006). Its creation resulted in the development of a general usage Artificial Intelligence Markup Language (AIML), which can be used to quickly create a basic bot from scratch. ALICE is a thrice winner of the Loebner Prize, an annual competition in artificial intelligence that awards chatbots judged to be the most human-like. The format of the competition is based on a standard Turing test (Shah, 2006).

Whilst it could be said that the ultimate goal regarding chatbots is to truly pass the Turing test (Turing, 1950) and convince a human judge that it (the bot) is human (Oppy and Dowe, 2016), our present focus is on using chatbots as conversational search/assistant interfaces. Instead of finding therapy content via the search box or menus, a chatbot offers an alternative search modality. At its simplest, a chatbot could guide the user in terms of disclosing emotions, therapy preferences and needs. Upon receiving the user's input, the bot could respond with some suggestions. User experience elements are critical as there are certainly cases where standard search interfaces/apps are a better, more effective choice than chatbots (Verber, 2016). There is however distinct potential in using chatbot searches. A conversational search mode can create a sense of connectivity and personalization that offers a uniquely effective way to collect input from users. Research suggests that users are more open and likely to share information, particularly on sensitive topics, when interacting with a machine interface (Weisband and Kiesler, 1996; Tantam, 2006; Lucas et al., 2014), thus it may be important to include this technology in mental health web-sites due to the stigmatizing and delicate nature of the topic.

Transforming search from a user-initiated task to a quasi-conversation can be more conducive to eliciting information from users regarding their wants and needs. Furthermore, chatbots could perform psychological assessment in a novel way and learn from user responses in real time. We could also tailor the way the suggestion is made relative to the user, as mentioned in the previous section. One other interesting aspect to consider is the extra secondary information that can be obtained from even a few input sentences from the user. As opposed to a static form with pre-defined responses or a search text box that receives one or two word inputs, the input gathered from a conversational search is going to have a richer structure. Linguistic analysis of this input could then contribute to assessing a user's immediate requirements as well as forming a part of an overall assessment of the user.

Beyond this, there are some other interesting usage possibilities in between a basic conversational search and a chatbot that is the equivalent of a human mental health professional. Whilst not yet being sophisticated enough to replicate a therapist, bots that can maintain a basic form of conversation beyond one-question, one-input, one-response are feasible and have been implemented for different purposes: as a virtual dietician for diabetic patients (Lokman and Zain, 2009); as an educational system for students (Mikic et al., 2009); and as an E-learning system for speech learning for disabled people (Bhargava and Nikhil, 2009) among others. The implementation of such a chatbot would mean that more conversational text could be collected from the user. A richer body of conversation to analyse presumably translates to the possibility of better content suggestions.

Also, more sophisticated conversational bots like this could be used to gather information about a user before they chat online with a real therapist. Whilst not yet activated on any of our trials, the MOST system does have a real-time client to human moderator online text chat feature. Upon requesting to chat, users are asked to complete a number of questions before being sent to a waiting queue until a moderator is ready to accept their request. The information that they fill in helps the moderator to assess the client and their priority. Rather than just being restricted to a set of rigid form questions, a chatbot could be used to converse with the client before the human moderator is available to accept their request. Upon doing so, the bot conversation text can be analyzed and used to provide the moderator with a pre-profile of the client with whom they are about to chat.

## Conclusions and future work

The innovative MOST system, which integrates online peer support and evidence-based interventions with a unique clinician and consumer-centered service delivery process, has thus far demonstrated its viability in a series of research trials. Given the data-driven opportunities afforded by the system and the aim to deploy it on a greater scale, an important next step in its evolution is the incorporation of advanced computational and artificial intelligence methods. This innovation will play a central role in furthering foundational goals of our interventions such as enhancing user engagement, facilitating the discovery and delivery of tailored therapy content and promoting autonomy, competence and relatedness. It will also serve to supplement, complement, or possibly even replace human moderators. Ultimately this endeavor will lead to a more scalable system that is better positioned to meet the unmet needs in large-scale mental health provision and long-term support.

A current focus of our work is on developing mechanisms to deliver individualized therapy suggestions based on linguistic analysis of newsfeed postings and other pertinent factors such as user preferences and histories. This is one specific example of how the analysis of user content can feed information retrieval services and is related to the more general field of using computational linguistic analysis to predict/determine psychological states and characteristics (Tausczik and Pennebaker, 2010; Gkotsis et al., 2016). As we move from controlled trials with a select group of participants each having the same established mental health condition to more general, publicly used sites where users will have a variety of conditions and are not pre-known trial participants, there is also the potential to deliver therapy content particularly relevant to a certain mental health condition based on analysis of a user's content (Bedi et al., 2015).

Apart from user content analysis, other possibilities include making suggestions based on geolocation data (Dredze et al., 2013) and self-monitoring/self-sensing data (Matthews et al., 2014). Regarding the former, one example would be to determine the geographical location or event of an active user using their mobile phone, retrieve an action that could make use of that location/event and deliver the suggestion via a mobile notification. Regarding the latter, one example would be to detect the onset of anxiety attacks with wrist sensor technology (Talbot, 2012; Kappas et al., 2013) and provide the user with appropriate therapy content. As discussed, we have also done some preliminary exploration into the implementation of therapy chatbots, which can simulate interactions with human beings to varying degrees of reality and will quite possibly become a mainstay of future e-mental health and help seeking web sites/applications.

## Author contributions

All authors materially participated in the research and/or article preparation and all authors have approved the final article. SD is the article's principal author and is responsible for the implementation of the software and creation of the underlying algorithms and data analysis. OS has contributed to work on the data and the writing/editing of the article. SR, GW, and RL provided conceptual guidance on the new methodology and study, critically reviewed the drafting of the manuscript, and were consulted when needed. CM contributed to the design and development of the main features covered in this paper. MA and JG contributed to the grant application, contributed with the conception and design of the project, conduct of the study, supervision and edits on the early and final draft.

## Funding

OS was supported via an Endeavour Research Fellowship. SR was supported via a Society for Mental Health Research Early Career Fellowship. MA was supported via a Career Development Fellowship (APP1082934) by the National Health and Medical Research Council (NHMRC).

### Conflict of interest statement

The authors declare that the research was conducted in the absence of any commercial or financial relationships that could be construed as a potential conflict of interest.

## Footnotes

^1^https://www.eheadspace.org.au/

^2^The posts shown in Figure 1 are actually real samples taken from the live Horyzons system.

^3^The IBM Alchemy service has been employed as it offers affordable expert text analysis capabilities accessibly, allowing us to straightforwardly implement our proof of concept. Limitations regarding its proprietary and unscrutinised nature will be something to consider. Short of conducting our own program into text analysis, academically reviewed alternatives include Valence Aware Dictionary and sEntiment Reasoner (VADER), Linguistic Inquiry and Word Count (LIWC), and SentiStrength. One advantage of some of these alternatives is that the software libraries could be hosted on our own servers.

^4^http://www.affectiva.com/
